# Teachers’ Patterns of Management of Conflicts With Students: A Study in Five European Countries

**DOI:** 10.5964/ejop.v16i1.1955

**Published:** 2020-03-03

**Authors:** Maria Clelia Zurlo, Federica Vallone, Elena Dell’Aquila, Davide Marocco

**Affiliations:** a Department of Political Sciences, University of Naples Federico II, Naples, Italy; b Department of Humanities, University of Naples Federico II, Naples, Italy; University of Wroclaw, Wroclaw, Poland

**Keywords:** conflict management, teacher’s conflict management styles, school, Europe, cluster analysis

## Abstract

The present study proposed an application of the Rahim’ Model of Conflict Management, and aimed to explore the styles of handling interpersonal conflicts with students adopted by teachers from five European countries (Italy, Spain, Germany, Belgium, Austria), identifying specific patterns and evaluating potential differences according to teachers' Gender, Age, Working Seniority and Country of belonging. Overall, 589 secondary school teachers completed a questionnaire consisting of Socio-demographic characteristics and the Rahim Organizational Conflict Inventory-II (ROCI-II, Form B). Non-hierarchical k-means cluster analysis was employed to derive patterns of conflict management, identifying four patterns labelled as Multi-strategic and Engaged, Multi-strategic and Solution-Oriented, Control-Oriented and Avoidant, and Mediating. Significant differences between countries were found in the numbers of teachers grouped across the four patterns. Findings identified stable and meaningful patterns for evaluating teachers' styles of management of interpersonal conflicts with students and for promoting teachers’ effectiveness in conflict management in the European school context.

In recent years, the growing changes of the modern educational context induced to emphasise the role of teachers and educators and the competences they are expected to possess in integrating knowledge with relational and technical skills (European Commission, 2013, 2017).

Indeed, teachers are constantly required to adapt their practices and to handle complexity, and classroom management represents one of their major responsibility not only in terms of planning, teaching new programmes, and organising the learning environment, but also in terms of managing relational aspects of the educational duties (García-Moya, Brooks, Morgan, & Moreno, 2015; Hagenauer, Hascher, & Volet, 2015), dealing with conflicts, and promoting positive relationships and a satisfactory class climate (Claessens et al., 2017).

In particular, nowadays, conflict resolution management represents one of the main challenges for teachers since conflicts with students from the new generation have become increasingly complex, and deriving not only from misunderstandings and disapproving behaviours during class, but also from perceived differences in worldviews, ideologies, cultures, and objectives (Causey, Thomas, & Armento, 2000; Doğan, 2016; Vasilescu, Popescu, & Popescu, 2012).

Therefore, a growing body of research explored teacher-students conflictual interactions (Allen et al., 2013; de Jong, van Tartwijk, Verloop, Veldman, & Wubbels, 2012), highlighting teachers' difficulties in conflict management and teacher-student negative interactions as playing a key role in determining a large number of outcomes both for teacher, in terms of perceived work-related stress and burnout (Evers, Tomic, & Brouwers, 2004; Spilt, Koomen, & Thijs, 2011; Zurlo, Iannario, & Piccolo, 2017; Zurlo, Pes, & Capasso, 2013, 2016), leaving intention (Zurlo & Pes, 2012) and attrition (Ingersoll & Smith, 2003), and for students, in terms of decreased engagement (Pianta, Hamre, & Allen, 2012), motivation, and performance (de Boer, Bosker, & van der Werf, 2010; Frymier & Houser, 2000).

Nevertheless, research also highlighted that the conflict has to be considered not only a source of pressure in teaching profession but also a significant opportunity for development and enhancement of skills, critical reflection, and growth at personal, interpersonal and group level (Doğan, 2016). Consequently, particular attention should be given not only to teachers’ ability to avoid conflict emergency but also to teachers' ability to adequately handle conflicts and to adopt constructive and creative management strategies without disrupting the educational goals and without affecting the relationship with students and the class harmony (Balay, 2006; Cornille, Pestle, & Vanwy 1999; Doğan, 2016; García-Moya, Brooks, & Moreno, 2019; Longaretti & Wilson 2006; Özgan, 2016).

Therefore, considering the significance of developing and enhancing teachers’ skills and competencies to successfully deal with potential conflictual situations that may emerge with students in the contemporary educational context, a deep and broad investigation of teachers’ way of handling conflicts is needed, also taking into account that there is still a lack of research conducted in the European Countries in this direction (European Commission, 2013).

## Rahim’ Model of Conflict Management Styles and Teacher-Student Interactions

In the field of organizational behaviour and management, a large body of research focused on conflict management (Thomas & Kilmann, 1974, 2008; Van de Vliert & Kabanoff, 1990), and one of the most adopted models of conflict management has been developed and proposed by Rahim and Bonoma (1979).

The Rahim’ Model of Conflict Management styles for interpersonal conflicts (Rahim, 1983a, 1983b, 2001; Rahim & Bonoma, 1979) aims at describing conflict and negotiation processes referring to two basic dimensions: concern for self (i.e., the degree to which individuals aim at satisfying their own concern in conflict management processes) and concern for others (i.e., the degree to which individuals attempt to accomplish with the concern of the other party involved in a conflict).

On the basis of different combinations of these two dimensions, five styles of handling interpersonal conflict have been distinguished: Integrating, Obliging, Dominating, Avoiding, and Compromising.

Integrating style indicates high concern both for self and for others and is characterised by a collaboration between the parties involved into the conflict with the aim to reach a mutual and acceptable solution through openness, exchange of information, examination and exploration of differences for arriving at a constructive solution that overwhelms personal and limited visions of the problem. This style is considered as a “Win-Win” strategy of conflict management, due to the emphasis given to the interest in optimizing rather than sacrificing for the other party.

Obliging style indicates a low concern for self and a high concern for others and is characterised by the recourse to a non-confronting behaviour aiming to minimise differences between the parties involved into the conflict by emphasising commonalities and sacrificing personal concern to safeguard the relationship.

Dominating style indicates high concern for self and low concern for others and is considered a “Win-Lose” strategy and a demonstration of authority or expression of a forcing behaviour aiming to win or defend one’s position often ignoring others’ needs and perspective.

Avoiding style indicates low concern both for self and for others, reflects an attitude to refuse to acknowledge and to openly face conflict and is often characterised by withdrawal, buck-passing, sidestepping situations.

Finally, Compromising style indicates intermediate concern for self and for others. This style involves splitting the difference, exchanging concession, or seeking a quick, middle ground position, whereby both parties involved in the conflict accept to give up something to reach mutually acceptable decisions.

In order to assess the amount of perceived organizational conflict at individual, group, and intergroup levels and the strategies adopted for handling conflicts, Rahim proposed two measurement tools, the Rahim Organizational Conflict Inventory I and II (ROCI- I & ROCI-II; Rahim, 1983a, 1983b, 2001), which assessed, respectively, perceived organizational conflict (ROCI-I) and the styles adopted for managing interpersonal conflict (ROCI-II).

In particular, the ROCI-II measures the styles of handling interpersonal conflict adopted by an organizational member to deal with his/her supervisor(s) (Form A), with subordinates (Form B), and with peers (Form C) (Rahim, 1983a, 1983b, 2001). The author made a clear distinction among the parties potentially involved into conflict (i.e., supervisor, subordinates, peers), considering that the adoption of one of the five styles within the negotiation process may significantly depend on the perceived symmetry/asymmetry of power within the relationship. In this direction, the author also highlighted that individuals may use all the suggested five styles of conﬂict management (Hart, 1991; Rahim & Bonoma, 1979; Thomas, 1977), and that the appropriateness of the use of the different styles is strongly influenced not only by the other party involved in the conflict, but also by the individual duties and goals, and by the specific characteristics of the conflictual situation (Rahim, 1986).

The application of the Rahim’ Model is of particular relevance within the school context. Indeed, teachers are constantly involved into negotiation processes, and their responses to potential conflicts with students are constantly influenced by the specific feature of teachers’ role as accountable for classroom management and by the different declinations of the dimensions of concern for self and concern for others within teacher-student interactions (i.e., Superior-Subordinates relationships). From this perspective, research highlighted that teachers may use different strategies to efficiently handle conflicts with students (Claessens et al., 2017; Doğan, 2016; Morris-Rothschild & Brassard, 2006), and they can recur to: Integrating strategies (e.g., reasoning with the student inside or outside the classroom setting; involving the student in individual and group settings to discuss the student’s behaviour); Compromising strategies (e.g., reasoning and discussing issues and problems with the student and/or with the whole class to explore new possible solutions and ways to deal with the individual and relational difficulties emerged); Obliging strategies (e.g., accommodating and/or deliberately ignoring minor disruptions or infringements); Avoiding strategies (e.g., delaying discussion and confrontation concerning individual and relational difficulties emerged; sending the student to the deputy principal); and, finally, Dominating strategies (e.g., issuing a verbal reprimand; asking student to withdraw from the class; requesting periods of school suspension).

The adoption of a specific style may also depend on the perceived role of the teacher as regulator and leader of the classroom environment, which may significantly influence its choices basing on its necessity to simultaneously achieve learning objectives and to assure a satisfactory group climate in the classroom.

In this direction, a study conducted by Doğan (2016) proposed an application of the Rahim’ Model (Rahim, 1983a, 1983b, 2001; Rahim & Bonoma, 1979) to explore conflicts occurring in school according to perceptions and views of teachers and the resolution strategies they adopt to deal with them. The study revealed that teachers were more frequently induced to adopt a dominating style to handle conflicts with students, due to their perception of the necessity to express their authority to efficiently managing conflicts with students of the new generation. Nonetheless, teachers also revealed to consistently use obliging and integrating styles due to the importance they attribute to find the best way to interact with students and to successfully solve the conflict by trying to understand their feelings, needs, and expectations. Moreover, the study revealed that teachers were also induced to adopt avoiding and compromising styles whenever they perceive the emergence of the conflict as harmful for the continuity and the quality of the teacher-student educative relationship.

In the same direction, a study conducted by Morris-Rothschild and Brassard (2006) also highlighted the importance of developing concern for student needs and perspectives to successfully manage conflicts and preserve a satisfactory classroom climate by adopting integrating, compromising and obliging strategies. Furthermore, the same study also underlined specific correlations between different styles of conflict management adopted by teachers, i.e., significant positive correlations between integrating, compromising, and obliging styles, significant positive correlations of avoiding style with both dominating and obliging styles, and significant negative correlations of dominating style with both integrating and compromising styles. These findings suggested the possibility to investigate a higher-order aspect of the Rahim' Model management styles to enlighten the presence of teachers’ specific patterns of handling conflicts in teacher-student interactions.

From this perspective, indeed, also research on conflict management suggested a broader approach that further insights into the Rahim’ Model, defining the negotiation process as resulting by the configuration of different styles rather than by the recourse to single and independent styles. In this direction, Rahim (Rahim, Antonioni, & Psenicka, 2001; Rahim et al., 2002) highlighted the possibility to reorganise and differently classify the styles for handling conflict according to the integrative (party's pursuit of own and others' concerns) and distributive dimensions (party's pursuit of own or others' concerns) which represented, respectively, the problem solving and bargaining strategies. Furthermore, with the same objective, a study conducted by Munduate, Ganaza, Peiro, and Euwema (1999) in a sample of managers identified five patterns profiles deriving from different combinations of strategies for handling conflicts, considered particularly useful to specifically investigate managers’ ways to successfully deal with conflictual situation that may arise with superiors and subordinates.

## Purpose of the Study

Taking into account the increasing complexity of the negotiation process within teacher-student conflictual interactions, the need for teachers to efficiently managing conflictual situations with students, and the recent research direction on conflict management, the present study aimed to apply the Rahim’ Model to explore teachers’ styles of handling interpersonal conflicts within teacher-student interactions and to identify specific patterns. Moreover, considering that research on conflict management also identified other dimensions that may play a significant role in influencing individuals’ negotiation process, such as gender (Blackburn, Martin, & Hutchinson, 2006; Gayle, Preiss, & Allen, 1994; Rahim, 1983a, 1983b, 2001), age, working seniority (Claessens et al., 2017; Morris-Rothschild & Brassard, 2006), and country of belonging (European Commission, 2013; Rahim et al., 2002), the study would also explore the distribution of subjects adopting the emerged conflict management patterns according to all these dimensions.

## Method

### Participants and Procedures

National online surveys were organized in five European countries (i.e., Italy, Spain, Belgium, Germany, and Austria) as part of the ACCORD Project (Attain Cultural Integration through COnflict Resolution skill Development; www.accord-project.eu) aiming to explore the styles of handling interpersonal conflicts adopted by secondary school teachers within teacher-student interactions. The questionnaire was made available online and widely diffused through partners’ teachers’ and secondary schools’ networks. All procedures performed in the present cross-sectional study were in accordance with the 1964 Helsinki declaration and its later amendments or comparable ethical standards. Overall, 589 teachers completed the questionnaire on a voluntary basis. Participants were fully informed about the aims of the study and about the confidentiality of the data. Every precaution was taken to protect the privacy of research subjects and the confidentiality of their personal information, and the questionnaires were anonymously completed. Health, dignity, integrity, and rights of participants were preserved, and data were collected with no physical and psychological hazard for research subjects.

### Measures

A questionnaire consisting of open and closed-ended questions to collect Socio-demographic characteristics (Gender; Age; Working Seniority; Country of belonging) and the Rahim Organizational Conflict Inventory-II (ROCI-II, Form B; Rahim 1983a, 1983b, 2001) was submitted online to secondary school teachers.

The Rahim Organizational Conflict Inventory-II (ROCI-II; Rahim 1983a, 1983b, 2001) consists of 28 items on a 5-point Likert scale, assessing the five styles of handling interpersonal conflict: Integrating (7 items, e.g., “I try to investigate an issue with my subordinates to find a solution acceptable to us”), Obliging (6 items, e.g., “I generally try to satisfy the needs of my subordinates”), Dominating (5 items, e.g., “I use my influence to get my ideas accepted”), Avoiding (6 items; e.g., “I attempt to avoid being “put on the spot” and try to keep my conflict with my subordinates to myself”), and Compromising (4 items, i.e., “I try to find a middle course to resolve an impasse”). A higher score represents greater use of a conflict management style. Moreover, considering the aim of the study to explore teachers’ styles of handling conflict within their interactions with students, the Form B of the questionnaire, assessing the interactions with subordinates (i.e., students) was adopted.

### Data Analysis

In accordance with the aims of the present study, all the analyses were carried out using the statistical software SPSS (Version 21).

Preliminarily, a number of Descriptive Analyses of all study variables were conducted and Pearson’s Correlations between the five conflict management styles were carried out.

Therefore, Cluster Analysis using a non-hierarchical k-means clustering procedure was applied to identify patterns of conflict management styles grouping teachers from the five countries, starting by establishing an initial partition, later calculating the means (centroid) of the clusters and determining the Euclidean distances to all the centroids in the clusters. The cases have been assigned to the nearest cluster, reducing the pooled within-group variance, and this procedure has been repeated until a solution was reached in which the clusters were stable (Aldenderfer & Blashfield, 1984). Indeed, the use of this iterative approach reduced the changes of biases entering the designation of initial cluster seeds, assuring stable clusters once the procedure reached the 2% convergent criterion (Aldenderfer & Blashfield, 1984; Hair, Anderson, Tatham, & Black, 1998; Lee, Lee, & Wicks, 2004).

Afterwards, a MANOVA and Tukey’s HSD post hoc test were used in order to validate the clusters solution, with the cluster membership as an independent variable and the five styles of handling conflict as dependent variables (Aldenderfer & Blashfield, 1984).

Finally, frequencies and percentages of subjects distributed across the emerged Conflict Management Patterns were calculated by Gender, Age, Working Seniority, and Country of belonging (Crosstabulations, Chi-square Analyses).

## Results

### Preliminary Analyses

Characteristics of study participants are displayed in Table 1.

**Table 1:** Characteristics of Study Participants (N = 589)

Characteristics	*n* (%)	*p*^a^
Gender		< .001
Male	173 (29.4%)	
Female	416 (70.6%)	
Age in years		< .001
Under 35	154 (26.1%)	
36-45	144 (24.5%)	
Over45	291 (49.4%)	
Working Seniority in years		< .001
Under 5	130 (22.1%)	
5-10	121 (20.5%)	
Over 11	338 (57.4%)	
Country		< .001
Austria	109 (18.5%)	
Belgium	116 (19.7%)	
Germany	42 (7.1%)	
Spain	213 (36.2%)	
Italy	109 (18.5%)	

^a^Differences were determined by Chi-square test.

Descriptive statistics (Mean scores and standard deviations) and Pearson’s Correlations between the five Conflict Management styles (Integrating, Avoiding, Obliging, Dominating, Compromising) are provided in Table 2. In particular, Integrating style revealed significant positive correlations with Compromising (*r* = .49, *p* < .01) and Obliging (*r* = .42, *p* < .01) styles and significant negative correlation with Dominating style (*r* = -.28, *p* < .01). Moreover, Compromising style revealed significant positive correlations with Integrating (*r* = .49, *p* < .01), Obliging (*r* = .38, *p* < .01) and Avoiding (*r* = .10, *p* < .05) styles and significant negative correlation with Dominating style (*r* = -.10, *p* < .05). Finally, it emerged that Avoiding style had significant positive correlations, on the one hand, with Obliging style (*r* = .12, *p* < .01), and, on the one other hand, with Dominating style (*r* = .15, *p* < .01).

**Table 2:** Descriptive Statistics and Pearson’s Correlations Between the Conflict Management Strategies

Conflict Management Strategies	*M*	*SD*	1	2	3	4	5
1. Integrating	4.12	0.56	1				
2. Obliging	3.38	0.51	.42**	1			
3. Dominating	2.52	0.71	-.28**	-.02	1		
4. Avoiding	2.80	0.74	-.02	.12**	.15**	1	
5. Compromising	3.66	0.61	.49**	.38**	-.10*	.10*	1

*p < .05. **p < .01.

### Cluster Analysis

Findings from non-hierarchical k-means cluster analysis suggested that a four-cluster solution was the most appropriate for the data, revealing four stable and meaningful patterns of conflict management adopted by teachers. In addition, results from MANOVA and Tukey’s HSD post hoc test confirmed the cluster solution, revealing that the four subgroups differed significantly on all conflict management styles, Wilk’s λ = .13, *F*(15, 589) = 113.34, *p* < .001, partial η^2^ = .49.

Table 3 shows the patterns identified, together with the values corresponding to the centroids of the styles for conflict management and the frequencies and percentages of the groups.

The first pattern (*n* = 136, 23.1%) represented teachers characterized by the lowest adoption of Avoiding style and by a moderate adoption of Integrating, Compromising, Obliging and Dominating styles. Therefore, the pattern was labelled as Multi-strategic and Engaged.

The second pattern (*n* = 143, 24.2%) consisted of teachers characterized by the highest adoption of Obliging, Avoiding and Compromising styles, but also by a relatively high adoption of Integrating and Dominating styles (displaying the second largest mean scores on these dimensions). Therefore, it was labelled as Multi-strategic and Solution-Oriented.

The third pattern (*n* = 153, 26.0%) included teachers reporting the highest adoption of Dominating style, a relative high adoption of Avoiding style (displaying the second largest mean score on this dimension), and the lowest adoption of Integrating, Compromising, and Obliging styles. Therefore, it was labelled as Control-Oriented and Avoidant.

Finally, the fourth pattern (*n* = 157, 26.7%) consisted of teachers characterized by the highest adoption of Integrating style and by a relatively high adoption of Obliging and Compromising styles (displaying the second largest mean scores on these dimensions), but also by a relatively low adoption of Avoiding style (displaying the second least mean score on this dimension) and by the lowest adoption of Dominating style. Therefore, it was labelled as Mediating.

**Table 3:** Conflict Management Patterns Centroids (N = 589)

Patterns	*n* (%)	*M* (± *SD*)				
		Integrating	Obliging	Dominating	Avoiding	Compromising
1. Multi-strategic and Engaged	136 (23.1)	3.98 (± 0.45)	3.16 (± 0.41)	2.65 (± 0.58)	2.08 (± 0.39)	3.35 (± 0.54)
2. Multi-strategic and Solution-Oriented	143 (24.2)	4.38 (± 0.38)	3.73 (± 0.43)	2.78 (± 0.64)	3.49 (± 0.59)	4.05 (± 0.43)
3. Control-Oriented and Avoidant	153 (26.0)	3.61 (± 0.46)	3.06 (± 0.39)	2.85 (± 0.60)	3.15 (± 0.42)	3.26 (± 0.48)
4. Mediating	157 (26.7)	4.50 (± 0.42)	3.58 (± 0.47)	1.85 (± 0.48)	2.45 (± 0.55)	3.97 (± 0.54)

### Gender, Age, Working Seniority, and Country of Belonging in Cluster Composition

Table 4 displays frequencies and percentages of subjects distributed across the Conflict Management Patterns emerged according to Gender, Age, Working Seniority, and Country of belonging. Findings from Chi-square Analyses revealed no significant differences in the number of subjects across the patterns with respect to Gender, Age and Working Seniority, while significant differences emerged with respect to the Country of belonging, χ^2^(12, 589) = 33.476, *p* < .001.

In particular, teachers from Austria (*n* = 109) were mainly represented in the Multi-strategic and Engaged pattern (*n* = 37, 33.9%) and in the Mediating pattern (*n* = 32, 29.4%), while they were under-represented in the Control-Oriented and Avoidant pattern (*n* = 18, 16.5%). Moreover, teachers from Belgium (*n* = 116) were over-represented in the Control-Oriented and Avoidant pattern (*n* = 39, 33.6%), and they were under-represented in the Multi-strategic and Solution-Oriented pattern (*n* = 18, 15.5%). Conversely, teachers from Germany (*n* = 42) were over-represented in the Multi-strategic and Solution-Oriented pattern (*n* = 16, 38.1%), and they were under-represented in the Mediating pattern (*n* = 5, 11.9%), while, conversely, teachers from Spain (*n* = 213) were mainly represented in the Mediating pattern (*n* = 61, 28.6%). Finally, teachers from Italy (*n* = 109) were mainly represented in the Multi-strategic and Solution-Oriented pattern (*n* = 34, 31.2%), and they were under-represented in the Multi-strategic and Engaged pattern (*n* = 13, 12.0%).

**Table 4:** Frequencies and Percentages of Subjects Across the Four Conflict Management Patterns by Gender, Age, Working Seniority, and Country of Belonging

Individual Characteristics	Multi-Strategic and Engaged	Multi-Strategic and Solution-Oriented	Control-Oriented and Avoidant	Mediating	Total	χ^2^	*df*	*p*
*n* (%)	136 (23.1)	143 (24.2)	153 (26.0)	157 (26.7)	589 (100)	-	-	-
Gender						5.099	3	.165
Male	33 (19.1)	46 (26.6)	53 (30.6)	41 (23.7)	173 (29.4)			
Female	103 (24.8)	97 (23.3)	100 (24.0)	116 (27.9)	416 (70.6)			
Age in Years						3.806	6	.703
Under 35	35 (22.7)	40 (26.0)	40 (26.0)	39 (25.3)	154 (26.1)			
36-45	37 (25.7)	33 (22.9)	42 (29.2)	32 (22.2)	144 (24.5)			
Over 45	64 (22.0)	70 (24.0)	71 (24.4)	86 (29.6)	291 (49.4)			
Working Seniority in Years						4.308	6	.635
Under 5	31 (25.6)	25 (20.7)	36 (29.7)	29 (24.0)	121 (20.5)			
5-10	72 (21.3)	83 (24.6)	86 (25.4)	97 (28.7)	338 (57.4)			
Over 11	33 (25.4)	35 (27.0)	31 (23.8)	31 (23.8)	130 (22.1)			
Country						33.476	12	< .001
Austria	37 (33.9)	22 (20.2)	18 (16.5)	32 (29.4)	109 (18.5)			
Belgium	31 (26.7)	18 (15.5)	39 (33.6)	28 (24.0)	116 (19.7)			
Germany	10 (23.8)	16 (38.1)	11 (26.2)	5 (11.9)	42 (7.1)			
Spain	45 (21.1)	53 (24.9)	54 (25.4)	61 (28.6)	213 (36.2)			
Italy	13 (12.0)	34 (31.2)	31 (28.4)	31 (28.4)	109 (18.5)			

*Note.* Differences were determined by Chi-square test. Reported values are *n* (%).

## Discussion

The present study aimed to test the hypothesis of the presence of different combinations of styles of handling conflicts adopted by teachers from five European Countries within teacher-student interactions and to identify specific patterns of conflict management, also exploring potential differences according to Gender, Age, Working Seniority and Country of belonging.

Firstly, the results of correlations and cluster analyses provided empirical evidence to support the proposition of investigating teachers’ behaviours in conflictual situations with students as configurations of different styles (Morris-Rothschild & Brassard, 2006; Munduate et al., 1999; Rahim et al., 2002).

Indeed, it emerged significant correlations among several pairs of conflict management styles adopted by teachers. In particular, Integrating style revealed significant positive correlations with Compromising and Obliging styles and significant negative correlations with Dominating style, indicating that, during a conflict episode with students, teachers may search integrating solutions and adopt in the same time compromising strategies (i.e., reasoning and discussing issues and problems with the student and/or with the whole class) and/or obliging strategies (i.e., accommodating and/or deliberately ignoring minor disruptions or infringements). Conversely, the recourse to dominating strategies (i.e., verbal reprimand; imposition of time-out/time-in; request of school suspension) appeared incompatible with the search of integrating solutions of the conflict.

In the same direction, also the Compromising style was significantly positively correlated with Integrating and Obliging styles and negatively correlated with Dominating style. Nonetheless, data highlighted that Compromising style was also positively correlated with Avoiding style (i.e., delaying discussion and confrontation concerning individual and relational difficulties emerged), suggesting that also the recourse to avoiding strategies may help, in some situations, to find a compromise. Conversely, the negative correlation between compromising and dominating strategies further indicates the relative incompatibility between these two styles of conflict management.

Finally, Avoiding style revealed significant positive correlations, on the one hand, with Obliging style, and, on the one other hand, with Dominating style, suggesting the possibility for teachers to recur to disengaged (i.e., Avoiding and Obliging styles) or authoritarian (i.e., Avoiding and Dominating) strategies for by-passing conflicts and misbehaviours within student-teacher interactions.

Hence, these preliminary results supported the possibility to investigate different configurations of styles of conflict management to identify specific teachers’ patterns of handling conflicts with students. Furthermore, the consideration that teachers tend to use Conflict Management Patterns rather than using independently different styles allow also to put further emphasis on the idea that successfully handling conflicts is strongly situation-specific (Rahim et al., 2002), and, consequently, that teachers may use different configurations of strategies to efficiently deal with and solve a conflictual situation with a student.

From this perspective, findings from cluster analyses revealed a four-pattern solution as the most suitable portrait, identifying four stable and meaningful patterns of handling conflicts with students that were substantially shared by teachers from the five different European Countries considered. The first pattern was labelled as Multi-strategic and Engaged and included teachers that, depending on the situation, displayed to be able to adopt different conflict management strategies except for the avoiding. Therefore, teachers grouped within the first pattern showed the tendency to be engaged with constancy in regulating and negotiating within the interactions with their students, and to be willing to find an effective way to solve the conflicts by reasoning and discussing with students, and, where it proves necessary, to adopt authoritative or accommodating strategies. Nonetheless, teachers characterized by this pattern profile also display difficulties in retreating from conflictual situations, always trying to face and solve them outright.

The second pattern was labelled as Multi-strategic and Solution-Oriented. It grouped teachers that showed to adopt all management strategies and that, consequently, were potentially able to efficiently handle a wide range of conflicts by searching for moment-to-moment solutions depending on the situation.

The third pattern was labelled as Control-Oriented and Avoidant and included teachers who mainly face the conflict by adopting both dominating and avoiding strategies, i.e., clearly defining and demonstrating their role and their authority, and directing the ways to handle the conflict taking predominantly into account concerns for self. Indeed, teachers within this pattern were also found to be less willing to adopt integrating, compromising and obliging strategies, often preferring to avoid the open confrontation with the student to protect themselves from the emergency or the escalation of conflictual situations.

Finally, the fourth pattern was labelled as Mediating, and it grouped teachers that reduced the recourse to dominating and avoiding strategies, and mainly adopted integrating, compromising and obliging strategies, reasoning with students and involving them in discussions aiming to promote negotiation processes.

Therefore, overall, these findings could be considered as useful to promote and support teachers in effectively and successfully dealing with conflicts by providing them with knowledge on the area of conflict management and by developing targeted feedback on teachers’ configuration of styles they primarily adopt for handling conflicts that may arise with students. In this perspective, it is necessary to further underline that there are no “right” or “correct” ways to manage conflicts and that each pattern may be adequate to efficiently handle conflictual interactions, but it may also hide some weakness that exposes teachers to potential risks (Evers, Tomic, & Brouwers, 2004; Rahim, 2001; Spilt, Koomen, & Thijs, 2011; Zurlo, Iannario, & Piccolo, 2017; Zurlo, Pes, & Capasso, 2013, 2016).

From this perspective, as regards teachers mainly adopting Multi-strategic and Engaged pattern of conflict management, on the one hand, they show to be able to recur to a wide range of strategies and to be always engaged, and these strategies should be considered as significant resources for a teacher to possess for achieving relational and educational goals. However, on the one other hand, they may also be at higher risk for work-related stress, due to their tendency to never avoid to openly and directly handle a conflict, spending, in some situations, an excessive amount of personal resources.

Conversely, as regards teachers adopting Control-Oriented and Avoidant pattern, their tendency to recur to more directing and authoritarian strategies in some cases could help quickly avoiding disruptions and, therefore, maintaining learning objectives. Nevertheless, they are also mainly induced to disengagement or imposing behaviours, and, consequently, they are exposed to risks to exacerbate conflicts, to increase students’ dissatisfaction and difficulties, and, potentially, to contribute creating a hostile class climate.

Furthermore, as regards teachers adopting Mediating pattern, the prevalent recourse to integrating, compromising and obliging styles revealed their high concern for the students’ needs and perspective. The recourse to this pattern should be considered as a significant resource for a teacher to achieve a satisfactory and stimulating class climate as they are always engaged in the educational relationship, encouraging challenging students to be involved in negotiation processes.

Finally, as regards teachers adopting Multi-strategic and Solution-Oriented pattern, their ability to recur to all the strategies depending on the situation can also be considered as a relevant resource to achieve optimal conflict management within the class. Nevertheless, the efficacy of this pattern depends on the development among teachers of adequate competencies to assess the situation, to identify the more adequate management style, and to implement the relational abilities for adopting this style. Consequently, this pattern induces to emphasise the importance of specific training to enhance teachers’ abilities to perceive and evaluate students’ needs as well as teachers’ relational competences.

In this perspective, the main interest of the present study was to identify common patterns of conflict management among different teachers, i.e., varying for gender, age, length of working experience and country of belonging.

Nonetheless, the study also aimed to explore potential differences across the four emerged patterns according to individual characteristics of teachers (i.e., Gender, Age, Working Seniority, and Country of belonging) in order to gain further indications on teachers’ predominant trends. Accordingly, the analysis of frequencies and percentages showed that patterns were adopted independently from gender, age and length of teaching experience, while it emerged specificities related to Country of belonging.

Nevertheless, in line with the Rahim’s perspective, the observed differences should be interpreted by considering teachers’ individual differences, the specific conflicting situations, and any potential specificity related to the national and European educational contexts. Indeed, these results could favour the reflection upon the possibility to develop more tailored interventions within European school context according to the pattern-specific needs, risks and resources.

For example, whilst teachers from Spain were significantly represented across the four pattern profiles, teachers from Germany and Italy seem to display a similar trend, revealing a higher tendency to use multiple strategies and to be solution-oriented; while, as opposed to teachers from Belgium, Austrian teachers showed to be less willing to adopt control-oriented and avoidant strategies.

Accordingly, interventions in these contexts should aim to promote greater balance in the ways they may handle conflicts with students by increasing the recourse to a wider set of conflict management strategies (by carefully assessing the specific conflicting situation), as well as the possibilities to also adopt directing/authoritarian strategies (when appropriate) in order to reduce the risk of exhaustion and burnout.

Furthermore, these results may raise the interest in exploring those individual and situational factors influencing these trends, deepening with teachers the different ways in which conflict management patterns can be concretely applied to the specific situations they may encounter in their work contexts.

Nevertheless, beyond the observed specificities, the present study provided four meaningful patterns useful to yield insight into the shared configuration of strategies for managing conflicts with students adopted by teachers from the five European Countries participating in the study, so maintaining the perspective of a common horizon while also observing potential differences in trends.

Despite the strengths of the present study, some limitations need to be underlined. Firstly, this research used a cross-sectional design, while longitudinal studies should be more adequate to determine the stability of identified patterns over time. Secondly, all the measurement tools were self-report, increasing the risk of social desirability bias. Thirdly, our findings identified four meaningful patterns of teacher’s conflict management styles, rising the interest to examine both individual and personality factors, as well as situational characteristics, such as types of students’ problematic behaviours and class climate, that may play a role in influencing the patterns of conflict management elicited in teachers. Moreover, further studies should be addressed to investigate individual and situational variables influencing the effectiveness of the adoption of each of the four patterns emerged in the resolution of conflictual interactions with students. Finally, findings revealing country specificities in conflict management pattern profiles suggested to further explore the role of cultural differences in the negotiation process adopting larger samples from other European countries.

Indeed, although the interest in providing country specificities in conflict management pattern profiles, that could be useful to rise and enhance awareness on the styles adopted by teachers in the European school context, findings from the present study should be interpreted with caution, considering the necessity to adopt larger samples from different countries to further explore and understand the effects of cultural differences on the model of conflict management strategies (Rahim et al., 2002).

In conclusion, the study provided empirical evidence for the identification of meaningful patterns of conflict management within teacher-student interactions, yielding useful tools to customise interventions aiming to promote teachers’ awareness on management strategies they adopt and to enhance their efficacy in regulating challenging interactions with students.

## Author Note

This publication reflects the views only of the authors, and the Commission cannot be held responsible for any use which may be made of the information contained therein.
